# Decoding the κ Opioid Receptor (KOR): Advancements in Structural Understanding and Implications for Opioid Analgesic Development

**DOI:** 10.3390/molecules29112635

**Published:** 2024-06-03

**Authors:** Zoe Li, Ruili Huang, Menghang Xia, Nancy Chang, Wenjing Guo, Jie Liu, Fan Dong, Bailang Liu, Ann Varghese, Aasma Aslam, Tucker A. Patterson, Huixiao Hong

**Affiliations:** 1 National Center for Toxicological Research, US Food and Drug Administration, Jefferson, AR 72079, USA; zoe.li@fda.hhs.gov (Z.L.); wenjing.guo@fda.hhs.gov (W.G.); jie.liu1@fda.hhs.gov (J.L.); fan.dong@fda.hhs.gov (F.D.); bailang.liu@fda.hhs.gov (B.L.); ann.varghese@fda.hhs.gov (A.V.); aasma.aslam@fda.hhs.gov (A.A.); 2 National Center for Advancing Translational Sciences, National Institutes of Health, Bethesda, MD 20892, USA; ruili.huang@nih.gov (R.H.); mxia@mail.nih.gov (M.X.); 3 Center for Drug Evaluation and Research, US Food and Drug Administration, Silver Spring, MD 20993, USA; nancy.chang@fda.hhs.gov

**Keywords:** opioid, receptor, structure, ligand, binding, mechanism, agonist, antagonist

## Abstract

The opioid crisis in the United States is a significant public health issue, with a nearly threefold increase in opioid-related fatalities between 1999 and 2014. In response to this crisis, society has made numerous efforts to mitigate its impact. Recent advancements in understanding the structural intricacies of the κ opioid receptor (KOR) have improved our knowledge of how opioids interact with their receptors, triggering downstream signaling pathways that lead to pain relief. This review concentrates on the KOR, offering crucial structural insights into the binding mechanisms of both agonists and antagonists to the receptor. Through comparative analysis of the atomic details of the binding site, distinct interactions specific to agonists and antagonists have been identified. These insights not only enhance our understanding of ligand binding mechanisms but also shed light on potential pathways for developing new opioid analgesics with an improved risk-benefit profile.

## 1. Introduction

The opioid crisis in the United States has escalated into a significant public health and policy concern, characterized by a rapid increase in opioid-related fatalities and a complex challenge for healthcare management. From 1999 to 2014, drug overdose deaths in the U.S. nearly tripled, with opioids accounting for a substantial proportion of these fatalities [1]. In 2014 alone, 60.9% of the 47,055 reported drug overdose deaths were opioid-related, a figure that rose to 63.1% of 52,404 deaths the following year [1,2]. Between 1999 to 2021, close to 280,000 Americans lost their lives due to overdoses related to opioids. (https://wonder.cdc.gov/, accessed on 12 March 2024) The count of drug overdose fatalities involving prescription opioids in 2021 surged to nearly five times the figure recorded in 1999. (https://wonder.cdc.gov/, accessed on 12 March 2024) This growing epidemic prompted the Centers for Disease Control and Prevention (CDC) to analyze drug overdose trends from 2010 to 2015, focusing on subgroups such as natural or semisynthetic opioids, heroin, methadone, and synthetic opioids other than methadone [1]. Despite a decrease in methadone-related deaths, attributed to regulatory efforts aimed at reducing its use for pain management, deaths from non-methadone synthetic opioids, largely driven by illicitly manufactured fentanyl, have surged [3,4,5]. The CDC’s findings underscore the urgent need for a comprehensive strategy that combines law enforcement and public health initiatives to address the multifaceted dimensions of the opioid crisis [6]. This approach necessitates targeted interventions to curb the rise of opioid misuse, enhanced surveillance and monitoring systems to track and understand the evolving nature of opioid-related deaths, and robust public health campaigns aimed at raising awareness and promoting safer prescription practices [6]. Moreover, it highlights the critical importance of advancing treatment options for opioid use disorder, including increasing access to medication-assisted treatment and supporting recovery services [6]. The opioid crisis not only poses a significant challenge to public health systems but also calls for a coordinated response that addresses the root causes of opioid addiction and implements effective prevention and treatment strategies. 

To effectively address the opioid crisis, while the strategies outlined by the CDC are useful, it is also essential to understand the underlying possible mechanisms of opioid actions. To understand how opioids work, researchers have employed pharmacological and biochemical methods to explore their signaling mechanisms. These studies confirmed that the primary action of opioids involves binding to their receptors, leading to the suppression of pain signals [7,8]. The foundation of opioid receptor research, dating back to the 1950s, culminated in the experimental confirmation of opioid receptors in 1973 through studies demonstrating the stereoselectivity of opioid binding [8,9,10,11,12,13,14]. This discovery set the stage for identifying multiple opioid receptor subtypes, including μ (MOR), δ (DOR), κ (KOR), σ, and ε, based on pharmacological experiments [15]. Subsequent advancements led to the isolation of cDNAs corresponding to MOR, DOR, and KOR, and the identification of a fourth receptor, the nociceptin opioid receptor (NOR), distinguished by its unique endogenous ligand, nociceptin/orphanin FQ [15,16]. 

Opioid receptors, localized in key areas of the brain and central nervous system involved in pain modulation and reward, play critical roles in inhibiting pain transmission and are implicated in reward and emotion processing [17,18]. The differential binding of endogenous opioid peptides to these receptors, such as endomorphins to MOR, dynorphins to KOR, and enkephalins to both MOR and DOR, highlights the complex interplay between various opioid peptides and receptors in regulating pain and reward pathways [7,19,20].

Understanding the mechanism of action of opioid receptors necessitated the elucidation of their structure, leading to the discovery that they belong to the class of G protein-coupled receptors (GPCRs) with a characteristic seven-transmembrane architecture [15]. This structure, confirmed through molecular cloning, hydropathy analysis, and X-ray crystallography, facilitates the coupling of opioid receptors with G proteins, triggering a cascade of intracellular signaling pathways upon agonist binding [15,21]. These pathways involve the inhibition of adenylyl cyclase, reduction in cAMP levels, and modulation of various ion channels, culminating in the physiological effects of the receptor [17,19,20,22]. This intricate signaling mechanism underscores the complex role of opioid receptors in cellular communication and response to opioids.

GPCRs engage in complex signaling pathways and can adopt diverse conformations influenced by ligand interactions, a phenomenon known as functional selectivity or ligand bias [23,24]. This principle, critical in opioid receptor functionality, allows for the selective activation of specific signaling pathways, including G protein-dependent, β-arrestin-dependent, and combined G protein-β-arrestin signaling [25,26,27]. G protein signaling attenuates cAMP levels, diminishes Ca^2+^ responses, and activates G protein-coupled inwardly rectifying potassium (GIRK) channels, while β-arrestin recruitment, following receptor phosphorylation, mediates receptor desensitization, internalization, and degradation, halting G protein signaling [28,29,30]. Additionally, β-arrestin can initiate other signaling pathways, such as mitogen-activated protein kinase (MAPK) and p38, and the interaction between Gαi subunits and β-arrestin can activate extracellular signal-regulated kinase (ERK), illustrating the opioid receptor’s intricate signaling dynamics and its therapeutic potential through biased and partial agonism [31,32].

**Figure 1:**
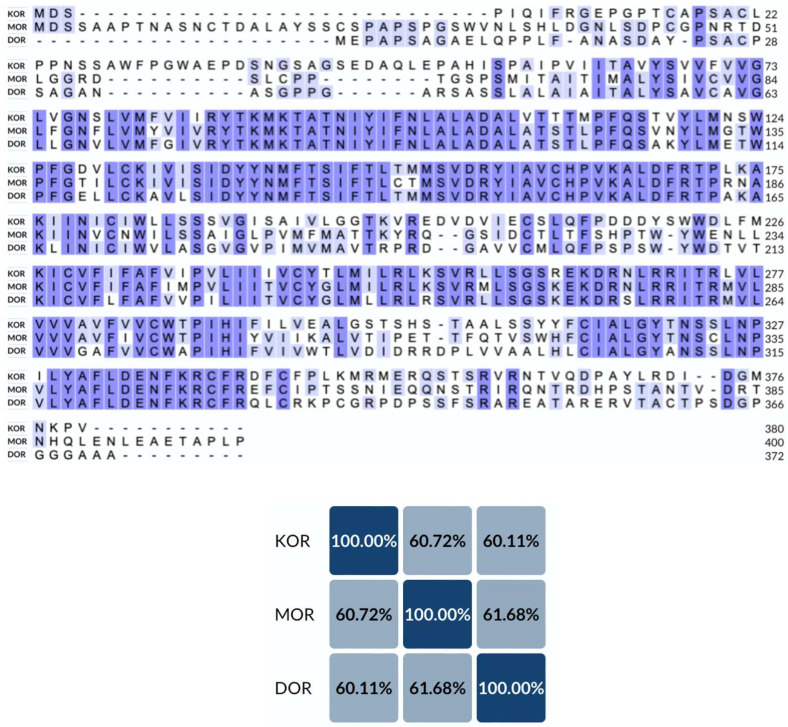
Sequence alignment result. The upper panel shows the sequence alignment of KOR, MOR, and DOR. The lower panel shows the percent identity matrix of the sequence alignment. Amino acids common to all subtypes are highlighted in dark purple, while those shared only by two subtypes are marked in light purple.

The different subtypes of opioid receptors have similar sequences, as the identity matrix revealed that the sequences of MOR, KOR, and DOR are approximately 60% similar (Figure 1). While the sequences and structural layouts of different subtypes of opioid receptors are very similar, they have different ligand specificity and pharmacological profiles. The endogenous peptides endorphins, enkephalins, and dynorphins bind to MOR, KOR, and DOR, but these peptides bind with various affinities and potencies. Specifically, β-endorphin demonstrates the highest potency as an endogenous ligand for the MOR, whereas enkephalins and dynorphins display increased affinity for DORs and KORs, respectively [33]. Another distinction among these opioid receptor subtypes is that, despite all inducing analgesia, MOR, KOR, and DOR agonists are positioned at different ends of hedonic continuums, as evidenced by pharmacological studies and genetic models [34]. In particular, MOR agonists induce euphoria and enhance stress management, KOR agonists evoke dysphoria, stress-related reactions, and negative emotional states, whereas DOR agonists alleviate anxiety and foster positive emotional states [34,35].

Extensive studies have been conducted to explore the biology, biochemistry, pharmacology, and mechanism of action of opioid receptors, aiming to deepen our understanding of these receptors [36,37,38,39,40,41,42,43,44,45,46,47]. This knowledge is crucial not only for addressing the opioid crisis but also for the development of novel therapeutic agents geared towards more effective pain management. While progress has been made in the development of agonists targeting opioid receptors, these compounds have encountered challenges in effectively mitigating the adverse effects associated with current opioid medications. 

Recent advancements in X-ray crystallography and cryogenic electron microscopy (cryo-EM) have significantly enhanced our ability to resolve protein structures, including those of opioid receptors. This increased understanding of three-dimensional protein structures has opened new avenues in drug design and development. These approaches include the identification of cryptic pockets on the receptor as potential binding sites, the exploration of allosteric binding sites for modulating receptor activity, the recognition of protein–protein interaction interfaces as potential drug targets, and the design of polypharmacy. Overall, three-dimensional protein structures offer valuable insights into the development of more targeted and efficacious treatments [48,49,50,51]. 

In this article, we focus on reviewing recently elucidated structures of the KOR, specifically analyzing its binding site and interactions with ligands.

## 2. Structural Overview of KOR

Various structures of the KOR have been reported since 2012 and are listed in Table 1. These include two structures bound with the antagonist JDTic (PDB ID: 6VI4, 4DJH) [52,53] and eight structures bound with various agonists. Among the agonist-bound structures, six are bound with small molecules MP1104 (PDB ID: 6B73) [54], nalfurafine (PDB ID: 7YIT) [38], GR89696 (PDB ID: 8DZR, 8DZS) [55], and momSalB (PDB ID: 8DZP, 8DZQ) [55]. The remaining three are bound with short peptides dynorphin (PDB ID: 7Y1F, 8F7W) and de novo cyclic peptide(DNCP)-β-naloxamine (NalA) (PDB ID: 8FEG) [56,57,58]. X-ray diffraction methods were employed in determining two antagonist-bound [52,53] and two agonist-bound structures [38,54]. Due to the challenge of stabilizing the receptor alone, the T4 lysozyme (T4L) fusion protein strategy and nanobodies were utilized to aid protein stabilization for crystallization. The remaining agonist-bound structures were obtained from cryo-EM experiments, with the receptors solved in complex with G proteins and/or megabodies (Table 1). In this review, we will focus on the structures bound with small molecules. The short peptide-bound structures are excluded from the analysis. For structures bound with the same ligand, only one structure is included in the analysis. The structures with PDB IDs 6VI4, 6B73, 7YIT, 8DZR, and 8DZP are included in the analysis.

**Figure 2:**
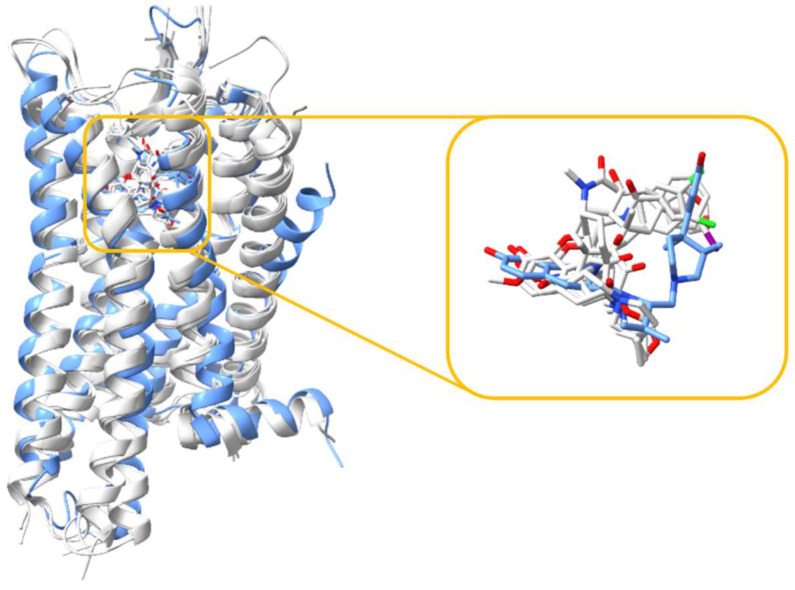
Alignment of five KOR structures, with one structure bound with an antagonist (depicted in blue) and the remaining four structures bound with agonists (depicted in white). The right panel shows an enlarged view, showcasing the alignment of all ligands, both the agonists and antagonist.

**Figure 3:**
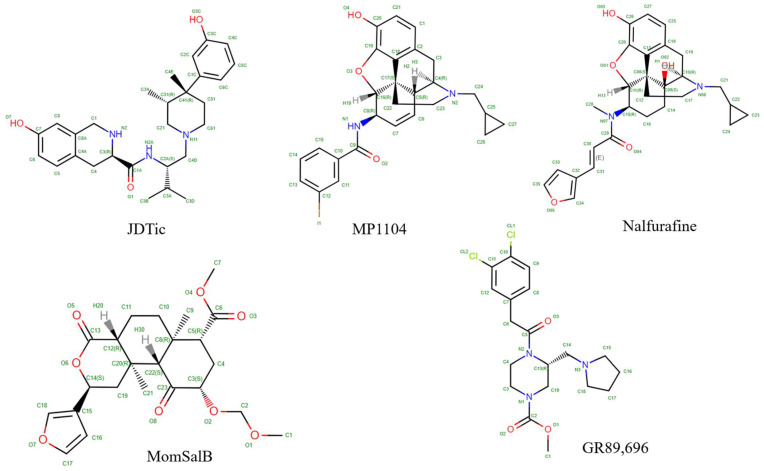
Two-dimensional structures of the ligands.

The KOR, like all other subtypes of opioid receptors including the MOR, comprises seven transmembrane helices interconnected by loops ECL1-3 and ICL1-3 [59]. The orthosteric binding site is located near the extracellular side of the helix bundle, and the alignment of the KOR structures demonstrated that all ligands bind within the same binding site (Figure 2). Both agonists and antagonists interact with the identical orthosteric site of the KOR, with minor structural variation observed. Figure 3 shows the 2D structures of the ligands. 

**Figure 4:**
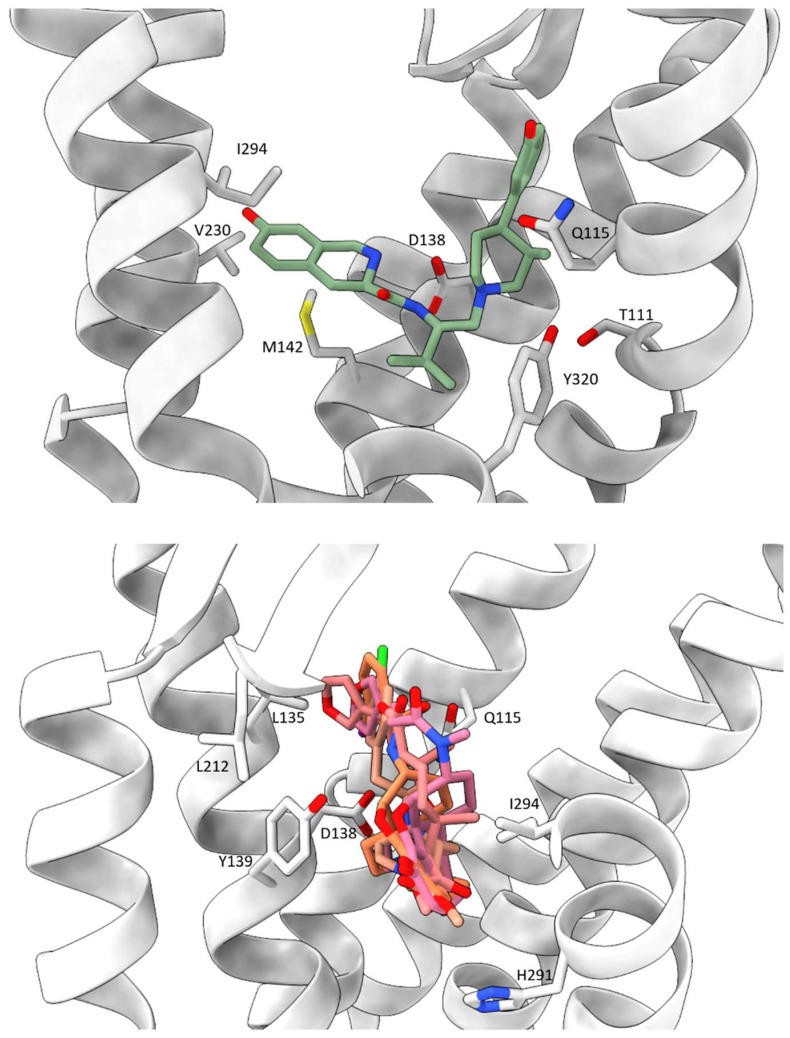
The ligand binding poses in KOR. The upper panel displays the antagonist binding pose and the lower panel showcases the agonist binding pose.

As for the binding of the antagonist JDTic, both the piperidine and isoquinoline moieties of the ligand’s protonated amines form salt bridges with the D138^3.32^ side chain (superscripts are Ballesteros–Weinstein numbering in GPCRs; all residue numbering scheme in this manuscript refers to the human KOR) (Figure 4) [53]. D138^3.32^ is conserved across all aminergic GPCRs, thereby playing a pivotal role in the selectivity of aminergic receptors towards ligands containing protonated amines [53]. Similarly, D138^3.32^ is conserved in all opioid receptors, and modeling and mutagenesis studies suggest its indispensable role in anchoring positively charged KOR ligands [53]. 

The amine moieties of the agonist MP1104 also form a salt bridge with D138^3.32^, albeit at a greater distance (3.0 Å) compared to the similar interaction observed between KOR and JDTic (2.6 Å), indicating a weaker ionic interaction (Figure 4) [54]. The phenolic groups of MP1104 extend towards TM5, establishing water-mediated hydrogen bonds with the backbone carbonyl oxygen of K227^5.39^, a phenomenon that is also observed in other opioid receptor structures [54]. Comparing to the isopropyl moiety in JDTic, the cyclopropylmethyl group of MP1104 extends deeper into a hydrophobic pocket located at the base of the orthosteric site. Various interactions between MP1104 and residues within this hydrophobic pocket were observed, including hydrophobic interactions between the cyclopropylmethyl group and the aromatic ring of the Y320^7.43^ side chain and with the side chain of W287^6.48^ and the backbone of G319^7.42^ [54].

Nalfurafine, another agonist with a morphinan scaffold, also forms a salt bridge interaction with the anchoring residue D138^3.32^. Adopting a reversed V-shaped binding pose akin to MP1104, nalfurafine establishes hydrogen bonds and hydrophobic interactions with the extracellular regions of the KOR. Additionally, similar to MP1104, the cyclopropyl methyl group of nalfurafine extends into a deeper pocket compared to the isopropyl group in JDTic (Figure 4) [38]. 

**Figure 5:**
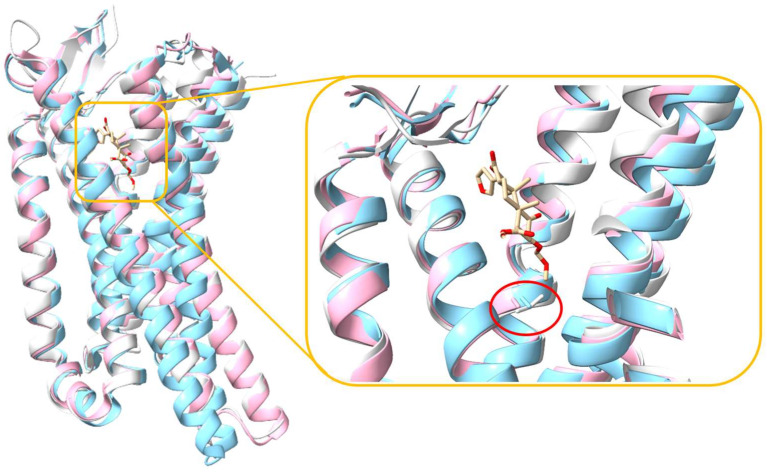
The momSalB-bound (colored in beige) KOR (PDB ID: 8DZP, colored in white) is aligned with agonist-bound MOR (PDB ID: 8EF6, colored in pink) and agonist-bound DOR (PDB ID: 6PT3, colored in light blue). The V108^2.53^ of KOR and the corresponding alanine in both DOR and MOR is represented in stick and circled in red.

Both momSalB and GR89,696 are potent and highly selective agonists of KOR. Although they occupy the orthosteric binding pocket of KOR, their core rings adopt different orientations, perpendicular to each other (Figure 4) [55]. Consequently, the ligands form distinct interactions with residues in their respective subpockets. Notably, mutations in the binding pocket have a greater impact on momSalB-mediated cAMP inhibition compared to GR89,696, likely due to the absence of anchoring interactions with D138^3.32^, making momSalB more susceptible to other residue contacts. Another significant distinction lies in the hydrophobic interactions formed by momSalB with specific residues contributing to its high potency, such as V108^2.53^, V134^3.28^, V230^5.42^, and I316^7.39^. Of particular interest is V108^2.53^, which has been identified as a determinant of ligand selectivity between KOR and MOR or DOR, as the latter two opioid receptors have alanine at the corresponding position [55]. Currently, there is no available structure of momSalB binding to MOR or DOR. To investigate the importance of V108^2.53^ to ligand selectivity, we aligned the momSalB-bound structure (PDB ID: 8DZP) [55] with the agonist-bound structures of MOR (PDB ID: 8EF6) [60] and DOR (PDB ID: 6PT3) [61] (Figure 5). We then measured the distances between momSalB and V108^2.53^ in KOR, as well as the corresponding alanine in MOR and DOR. The distances were found to be 3.7 Å for momSalB and V108^2.53^ in KOR, 4.5 Å for momSalB and A98^2.53^ in DOR, and 4.8 Å for momSalB and A119^2.53^ in MOR. The shorter distance between momSalB and V108^2.53^ in KOR compared to the other opioid receptor subtypes may explain momSalB’s selectivity for KOR.

**Figure 6:**
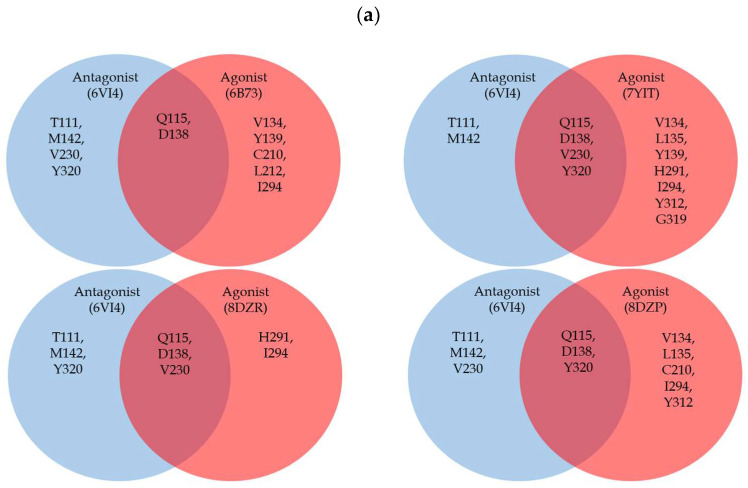

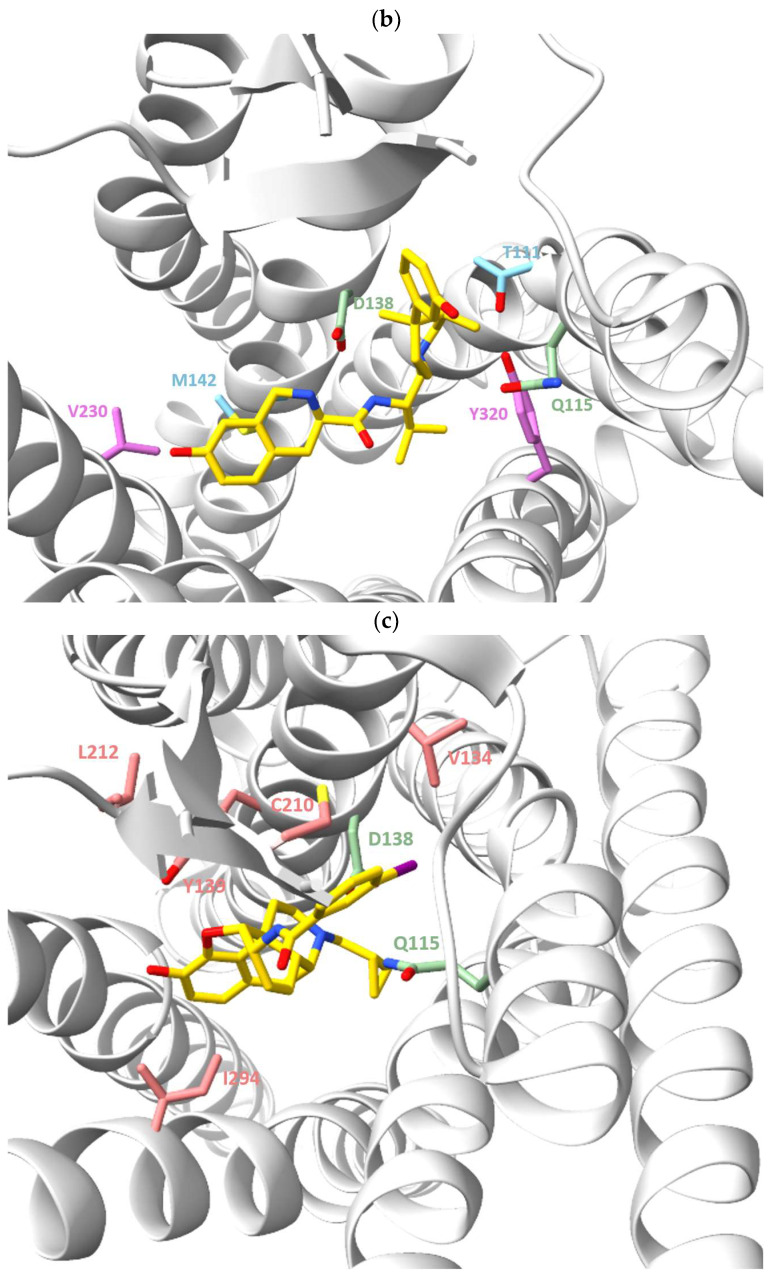

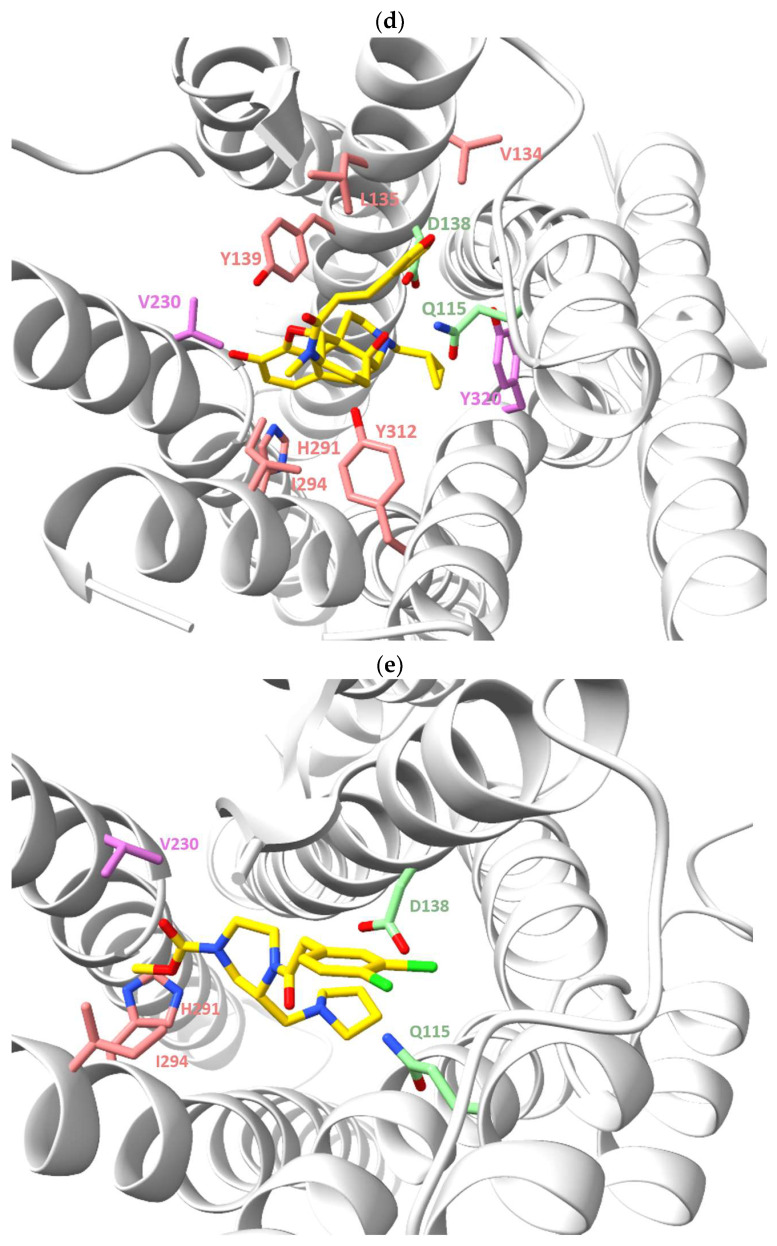

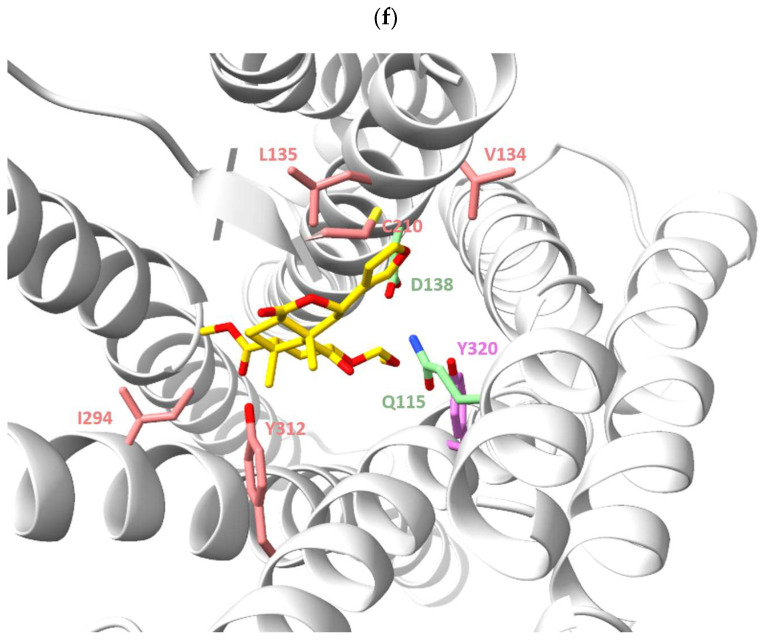
(**a**) Venn diagram showing the common and distinct residues found within 3.5 Å for antagonist (depicted by the blue circles) and agonist (depicted by the red circles). (**b**) Residues within 3.5 Å for antagonist JDTic (PDB ID: 6VI4): the residue unique to antagonist is colored in light blue, residue common to both agonist and antagonist is colored in light green, and the residue that is partially shared between agonist and antagonist is colored in light purple. (**c**–**f**) Residues within 3.5 Å for agonist MP1140 (PDB ID: 6B73), nalfurafine (PDB ID: 7YIT), and GR89696 (PDB ID: 8DZR), momSalB (PDB ID: 8DZP) respectively. The residue unique to agonist is colored in light coral, residue common to both agonist and antagonist is colored in light green, and the residue that is partially shared between agonist and antagonist is colored in light purple. All ligands are colored in yellow.

Despite the overall structural resemblances among KOR structures, certain unique aspects of ligand binding may lead to potential disparities in the binding pockets. To address this, our initial analysis focused on the composition of surrounding residues within 3.5 Å of the ligands across all structures. This examination revealed a range of 5 to 11 residues within this proximity, with at least half being hydrophobic and the remainder comprising charged or polar residues (Table 2). This analysis indicated a high similarity in the residue composition surrounding the ligands. To further explore this, we categorized the residues based on ligand types, aiming to discern whether certain residues were shared between agonists and antagonists or unique to one type of ligand. We refined our search by limiting the distance between the ligand and the receptor to 3.5 Å. Since there is only one antagonist-bound structure, it is compared to each agonist-bound structure individually, as depicted in Figure 6. The residues shared between agonist and antagonist include Q115^2.60^ and D138^3.32^. Residues unique to the antagonist are T111^2.56^ and M142^3.36^, while those unique to the agonist comprise V134^3.28^, L135^3.29^, Y139^3.33^, C210^45.50^, L212^45.52^, H291^6.52^, I294^6.55^, Y312^7.35^, and G319^7.42^.

To delve deeper into the analysis of the binding site, we computed the solvent-accessible surface area (SASA) and solvent-accessible volume (SAV) for each structure. While residues proximal to the ligand maintain similar conformations, ligand binding may lead to conformational changes in residues within the second interaction shell. Consequently, different ligands binding to the same site could result in subtle differences in the solvent-accessible surface area and volume of neighboring residues. Table 2 provides the SASA and SAV values for all structures, calculated using CASTp 3.0 [62]. 

**Figure 7:**
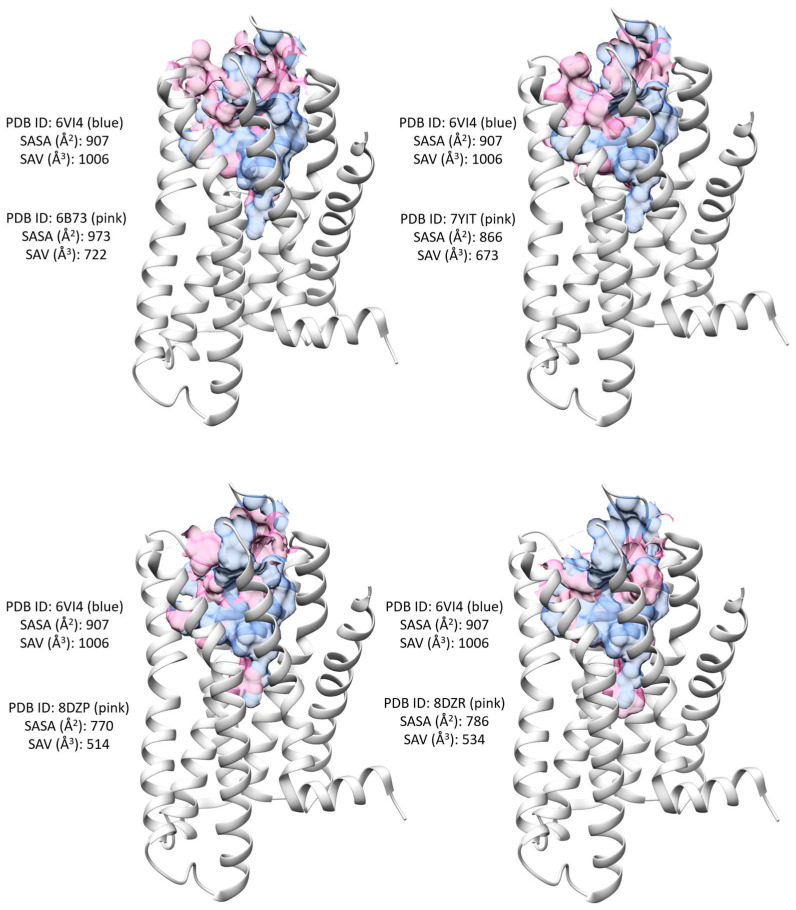
Comparison of the solvent-accessible volume (SAV) in the antagonist-bound structure (SAV depicted in blue) with that in the agonist-bound structures (SAV highlighted in pink).

Notably, no significant difference was observed in the SASA across all structures. However, the antagonist-bound structure exhibited the largest SAV compared to the agonist-bound structures (Figure 7). This observation aligns with the smaller orthosteric binding site seen with agonists across class A GPCRs and reflects the conformational changes that occur during the inactive to active transition. Despite both agonists and antagonists binding to the identical site, variations in ligand size contribute to discrepancies in the shape complementarity of the binding pocket. This observation could be a result of either conformational selection or induced-fit mechanism.

**Figure 8:**
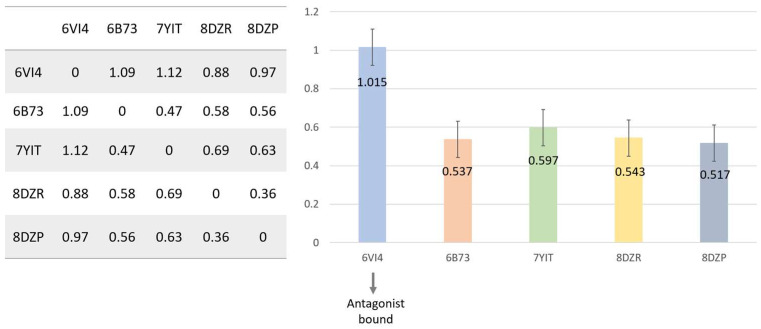
Comparison of the binding pocket RMSD between the antagonist-bound and agonist-bound structures. The left panel lists RMSD for all pairs of the structures. The right panel gives RMSD for each of the five structures aligned with the other four structures. The average of the four RMSD values for each of the five structures is plotted as a bar, and the corresponding standard deviation is presented by the stick on the bar. Structures of apo KOR were not included in the comparison, as our interest is to examine the difference between agonist-binding pocket and antagonist-binding pocket.

We conducted an analysis of the root mean square deviation (RMSD) of the binding pocket between the antagonist-bound structure and each of the agonist-bound structures, as well as the RMSD between agonist-bound structures. The average RMSD between antagonist-bound structure and agonist-bound structures is 1.015 Angstrom, notably higher than the average RMSD between agonist-bound structures, which was approximately 0.5 Angstrom (Figure 8). This discrepancy suggests a distinct binding site for the antagonist-bound structure compared to the agonist-bound structures, while the agonist-bound structures exhibit a high degree of similarity in their binding pockets.

Molecular dynamics simulation is a widely utilized technique for studying protein dynamics at the atomic level, facilitating a comprehensive understanding of small molecule binding and the mechanisms underlying protein functional selectivity [63,64,65,66]. Multiple studies have employed molecular dynamics simulations to explore various aspects of the KOR, including ligand binding and selectivity, the activation mechanism, and biased signaling [38,67,68]. In this review, we consolidate the findings pertaining to how ligand binding induces distinct receptor conformations and the mechanisms underlying ligand selectivity. 

An et al. investigated conformational dynamics and equilibria of the KOR upon binding agonist MP1104 and antagonist JDTic. They simulated the apo forms starting from active and inactive crystal structures, revealing that apo KOR is the most stable state with an open pocket, while active apo readily relaxes to inactive conformations [68]. Agonist binding shifts the conformational equilibrium towards the active state but is insufficient to fully stabilize it compared to KOR bound to both agonist and the stabilizing nanobody. In contrast, antagonist binding allows flexibility of the intracellular domain of KOR but keeps the inactive conformational equilibrium intact. This demonstrates that KOR does not act as a simple on/off switch upon ligand binding but rather samples multiple distinct conformations, including active, inactive, and intermediate states. Agonist binding biases the receptor towards the active state, while antagonist binding preserves access to the inactive and intermediate states. Specific residues D138^3.32^, N141^3.35^, and W287^6.48^ were observed to change rotameric conformational states between the active and inactive forms. The bulkier agonist disrupts the inactive states of these residues due to steric clashes, while the slimmer antagonist keeps them intact. This study offers insight into how agonists and antagonists influence the behavior of the KOR, providing valuable insights into receptor conformations that are not easily discernible in static structures. 

Saleh et al. compared the binding of JCTic and Alvimopan to the KOR to understand the molecular basis for their differing selectivity. JDTic and Alvimopan share a common scaffold based on the trans-3,4-dimethyl-4-(3-hydroxyphenyl)piperidine pharmacophore, which confers non-selective opioid receptor antagonism [67]. However, modifications to this scaffold have resulted in JDTic being highly KOP-selective, while Alvimopan is selective for the MOR. Their study has found that JDTic showed more limited conformational change over the course of the molecular dynamics simulation compared to Alvimopan. It maintained interactions with key residues for antagonism and a set of residues unique to KOP that form a hydrophobic “selectivity pocket” (V108^2.53^, V118^2.63^, I294^6.55^). An additional interaction with E297^6.58^, previously shown to confer selectivity in morphinan antagonists through the “message-address concept”, was also seen. In contrast, Alvimopan shifted its pose over the course of the simulation, losing an initial polar interaction with E297^6.58^ and interactions with V108^2.53^ and V118^2.63^, while maintaining contacts with residues common to antagonism across opioid receptor types. Overall, maintaining the specifically bound pose, enabled by JDTic’s V-shaped structure interacting with KOP residues D138^3.32^, W287^6.48^ and the selectivity pocket, appears key for high selectivity.

## 3. Future Perspective

The elucidation of high-resolution structures of the KOR, particularly in complexes with agonists and antagonists, provides invaluable insights into the molecular mechanisms underlying ligand binding and receptor activation. Researchers can employ rational drug design strategies to develop novel compounds for pain management and addiction. However, translating these structural insights into clinical solutions poses several potential challenges. While high-resolution structures offer detailed snapshots of the receptor–ligand interactions, translating this knowledge into clinically viable therapeutics requires overcoming obstacles such as pharmacokinetic properties, off-target effects, and drug delivery strategies. Additionally, the complexity of opioid receptor signaling pathways and the interplay between different receptor subtypes may present challenges in developing selective and efficacious drugs that specifically target the KOR without affecting other opioid receptors. 

Furthermore, the current limited availability of KOR structures may not provide sufficient details to fully understand the intricacies of agonist and antagonist binding. To address this limitation, it is crucial to obtain more antagonist-bound structures, as these can offer complementary insights into the conformational dynamics of the receptor in both active and inactive states. By expanding the structural database of the KOR, researchers can gain a more comprehensive understanding of the ligand-binding landscape and the structural basis of receptor activation and inhibition. 

In conclusion, the future of KOR-targeted drug discovery and development may hold great promise, driven by advancements in structural biology and computational techniques. The growing understanding of the structural aspects of the receptor and the increasing number of KOR structures can pave the way for the development of safer, more selective, and efficacious therapeutics for pain management and addiction treatment.

## Figures and Tables

**Table 1:** List of available structures of kappa opioid receptor and details regarding the structures.

PDB ID	Ligand	Ligand Type	Resolution	Complex with	Method	Reference
6VI4	JDTic	antagonist	3.3	Nanobody 6	X-ray	[52]
4DJH	JDTic	antagonist	2.9	Lysozyme	X-ray	[53]
6B73	MP1104	agonist	3.1	Nanobody	X-ray	[54]
7YIT	nalfurafine	agonist	3.3	Nanobody 39	X-ray	[38]
8DZR	GR89696	agonist	2.6	G alpha gustducin protein, Gβ-1, Gγ-2, scFv16	Cryo-EM	[55]
8DZS	GR89696	agonist	2.7	Guanine nucleotide-binding protein G(z) subunit alpha, Gβ-1, Gγ-2, scFv16	Cryo-EM	[55]
8DZP	momSalB	agonist	2.7	Gα_i_-1, Gβ-1, Gγ-2, scFv16	Cryo-EM	[55]
8DZQ	momSalB	agonist	2.8	Guanine nucleotide-binding protein G(o) subunit alpha, Gβ-1, Gγ-2, scFv16	Cryo-EM	[55]
7Y1F	dynorphin	agonist	3.3	Gα_i_-1, Gβ-1, Gγ-2, scFv16	Cryo-EM	[56]
8F7W	dynorphin	agonist	3.2	Gα_i_-1, Gβ-1, Gγ-2, scFv16	Cryo-EM	[57]
8FEG	De Novo Cyclic Peptide (DNCP)-β-naloxamine (NalA)	agonist	2.5	Gα_i_-1, Gβ-1, Gγ-2, scFv16	Cryo-EM	[58]

**Table 2:** List of available structures of the kappa opioid receptor, along with the detailed composition of binding pocket residues for each structure. Data for all residues within 3.5 Å of the ligand are included, with a breakdown of the number of charged, polar, and hydrophobic residues. The binding site solvent accessible surface area and solvent accessible volume are also provided.

PDB ID	6VI4	6B73	7YIT	8ZDR	8DZP
Ligand	JDTic	MP1104	nalfurafine	GR89,696	momSalB
Ligand type	antagonist	agonist	agonist	agonist	agonist
Charged residues	1	1	2	2	1
Polar residues	2	2	4	1	3
Hydrophobic residues	3	4	5	2	4
Binding site SASA (Å^2^)	907	973	866	770	786
Binding site SA volume (Å^3^)	1006	722	673	514	534
